# Lipid Nanoparticles Carrying Essential Oils for Multiple Applications as Antimicrobials

**DOI:** 10.3390/pharmaceutics17020178

**Published:** 2025-01-31

**Authors:** Elenice Francisco da Silva, Fernanda Aparecida Longato dos Santos, Henrique Machado Pires, Luciana Machado Bastos, Lígia Nunes de Morais Ribeiro

**Affiliations:** 1 Institute of Biotechnology, Federal University of Uberlandia, Uberlandia 38405-302, Brazil; lucianabastos.ufu@gmail.com; 2 School of Veterinary Medicine, Federal University of Uberlandia, Uberlandia 38402-018, Brazil; fernanda.longato60@gmail.com; 3 Vaccine Development Laboratory, Butantan Institute, São Paulo 05503-900, Brazil; henrique.pires.esib@esib.butantan.gov.br

**Keywords:** lipid nanoparticles, essential oil, antimicrobial activity

## Abstract

Lipid nanoparticles (LNPs) are versatile delivery systems with high interest because they allow the release of hydrophobic and hydrophilic molecules, such as essential oils (EOs) and plant extracts. This review covers published works between 2019 and 2024 that have reported the use of essential EO-based LNPs with antimicrobial properties and applications in human and animal health, as well as biopesticides. In the human healthcare field, reports have addressed the effect of encapsulating EOs in lipid nanosystems with antiviral, antibacterial, antiprotozoal and antifungal activities. In animal care, this still needs to be more deeply explored while looking for more sustainable alternatives against different types of parasites that affect animal health. Overall, the antibacterial activities of LNPs carrying EOs are described as alternatives to the use of synthetic antibiotics. In the field of agriculture, studies showed that these approaches in the control of phytopathogens and other pests that affect food production. There is a growing demand for innovative and more sustainable technologies. However, there are still some challenges to be overcome in order to allow these innovations to reach the market.

## 1. Introduction

Nanotechnology is an innovative field with many applications in the pharmaceutical, cosmetic, food and agricultural industries [1]. The nanoencapsulation of active agents is one of its most successful applications, which is designed to improve their functional performance and increase the stability and bioactivity due to a sustained bioactive release profile [2]. In the farming field, it has gained relevance in pesticides, nanofertilizers and nanoremediation. It produces several benefits, such as a reduction in the agrochemical concentration in formulations and water contamination, improvement of crop development and soil quality, and decrease in occupational risks [3]. Undoubtedly, nanotechnology is consolidated in the biomedicine field. It represents a paradigm shift in healthcare strategies. The industry, clinicians, patients, regulatory authorities and governments have to be prepared to explore such novel technologies as efficient and safe treatments and theranostics in order to improve the life quality of humans and other animals [4].

In particular, lipid nanoparticles (LNPs) are emerging as promising alternatives for active molecule delivery systems development, mainly due to their biocompatibility and ability to encapsulate hydrophobic and hydrophilic molecules, in addition to enabling the use of biodegradable and cheap excipients [2,5,6].

However, there are several translational challenges to be overcome before nanomedicines can be marketed. Thus, considering the multidisciplinary expertise of the university, industry and clinical-based researchers, the DELIVER framework (D—defined target product profile; E—essential characterization; L—lead candidate optimized; I—intellectual property patented; V—validated efficacy and safety; E—economical and scalable; R—regulatory and clinical pathway) was recently proposed [4]. This framework comprises a set of criteria that must be considered during the early stage of nanodrug development. It can help to enable the clinical success, regulatory approval and market registration of nanodrugs in the near future. This report also suggests the use of biocompatible and biodegradable nanomaterials, merging the desired efficacy with biocompatibility, stability and manufacturability [4]. Furthermore, specifically regarding agricultural ecosystems, it is essential to explore the use of biodegradable and ecofriendly nanomaterials that promote plant growth and metabolism, minimizing the environmental risks [3].

In this sense, the nanoencapsulation of bioactive compounds (e.g., EO) has garnered huge interest in nanomedicine and agrochemical development, while also considering the sustainability and low-cost production issues [2,7,8]. In the last few years, many strategies have been explored in terms of LNP, polymer-based nanocapsule/nanoparticle and cyclodextrin developments [6]. In here, we focus the discussion on lipid-based nanosystems, such as nanoemulsions (NE), liposomes (LUV), niosomes (NIO), solid lipid nanoparticles (SLN) and nanostructured lipid carriers (NLC).

LUV were the first lipid-based nanoformulations developed. They are spherical micro or nanovesicles consisting of one or more phospholipid bilayers surrounded by an internal aqueous compartment, which enables the encapsulation of hydrophobic, hydrophilic and amphiphilic molecules [9,10]. In addition, cholesterol is commonly added to the formulations to decrease the fluidity of unsaturated fatty acids, increasing the permeability of hydrophobic drugs [11]. There are several preparation methods, with lipid film hydration being the most used [12]. LUV are biocompatible and non-immunogenic. However, the high-cost production and the limited physicochemical stability usually restrict their applications [1,5,13].

NIO are comparable with LUV in terms of their structural organization in mono- or multilayer spherical vesicles composed of amphiphilic molecules, showing particle sizes in the range of 10–1000 nm [14]. On the other hand, NIO are based on non-ionic surfactants, and cholesterol and can encapsulate hydrophilic and hydrophobic molecules in its structure [15]. In comparison with LUV, NIO are more physiochemically stable, offering benefits such as low-cost production and a higher shelf life [16]. There are several well-determined NIO preparation methods, including thin-film hydration, ether injection, reverse-phase evaporation, microfluidization, supercritical carbon dioxide fluid, transmembrane pH gradient, multiple membrane extrusion, heating and bubble techniques [14,15].

NE are nanostructured colloids composed of water and oil and stabilized by a surfactant and cosurfactant [17]. This nanosystem is kinetically stable and the particle sizes typically range from 20 to 200 nm [9,18]. NE have some advantages compared with other colloidal systems, such as a simple and low-cost preparation process and physical and thermodynamic stability [5]. The most used techniques to produce NE can be categorized into high-energy (high-pressure homogenization, microfluidization and ultrasonication) and low-energy (phase inversion/composition and emulsification) methods [18].

SLN are considered the first lipid nanoparticles generation and were reported for the first time in 1990. They have emerged as a promising alternative to LUV in order to overcome their previously mentioned disadvantages. In general, SLN are composed of individual or a mixture of solid lipids at room and body temperatures, plus an aqueous phase containing a surfactant [13]. Different types of lipids can be used, such as triglycerides (trimyristin, tripalmitin), fatty acids (stearic acid, oleic acid) and waxes (cetyl palmitate, beeswax). Its lipid content represents approximately 0.1–30 (%, *w*/*w*), while the surfactants are generally used in concentrations of 0.5–5% [13]. On the other hand, NLC are the second lipid nanoparticles generation and are based on a lipid structural matrix composed of a mixture of solid and liquid lipids at both body and room temperatures, stabilized by a surfactant [19]. They were developed in order to solve some limitations of SLN, such as the low capacity for loading molecules and the expulsion of the active compound during the storage period. NLC have a higher capacity to encapsulate hydrophobic molecules compared with SLN due to the imperfect crystalline structure of the lipid matrix. In addition, it offers high drug stability, excellent bioavailability, safety, a low production cost and scale-up ability [20,21,22]. The most common production methods for SLN and NLC include high-pressure homogenization (hot and cold), microemulsification, solvent emulsification (evaporation or diffusion), solvent injection, double emulsion, ultra-sonication or high-speed homogenization, spray drying and microfluidics techniques [23].

Lipid-based nanosystems can provide a flexible platform for dermal and transdermal drug delivery and vaccine developments [9,24], such as Spikevax^®^ and Comirnaty^®^ indicated for COVID-19 immunization [25,26]. Moreover, the specific structure of LNP provides a safe and stable structure to carry mRNA and other vaccine components [27]. The liposomal and LNP systems are among the main technologies used in the development of drugs recently approved by the Food and Drug Administration (FDA) [25], in addition to synthetic molecules [7].

In this sense, essential oils (EO) are natural sources of therapeutic molecules. EO come from the secondary metabolism of plants and can be extracted from different structures: bark, leaves, flowers, buds, seeds, roots and fruits. They are complex mixtures of bioactive compounds derived from aromatic plants and are primarily composed of volatile terpenes and terpenoids [28]. Several biological properties have been described, including antimicrobial, antioxidant, analgesic, anti-inflammatory, anti-cancer and neuropharmacological effects, as well as repellant, larvicidal and biopesticidal effects [6,28,29,30,31]. In addition, they are Generally Recognized as Safe (GRAS) by the FDA and the Environmental Protection Agency (EPA), offering low risk to the environment, animals and humans, making them applicable in the medicine, agriculture, food industry and cosmetics fields [12,18]. However, there are some properties that limit their industrial application such as toxicity, low solubility and physicochemical stability, and high volatility and hydrolysis capacity [2]. In order to overcome such limitations, the nanoencapsulation of EO by LNP is considered a versatile alternative. Several systems have been successfully developed for multipurpose uses [19,22,32,33,34,35].

This review provides an overview of lipid-based nanosystems encapsulating EO with human, veterinary and environmental applications. The applied methodology focused on the selection of works published between 2019 and 2024 that provide data regarding the structural characterization of nanoparticles, physicochemical stability, safety and efficacy assays against microorganisms with interest in the human, veterinary and environmental fields. 

## 2. Application of Lipid Nanoparticles Loading Essential Oils as Antimicrobial Agents

LNP are considered the pioneers in nanostructured drug delivery systems. They were the most reported nanoparticles in clinical trials from 2016 to 2021. LUV were the most described, with 44% of the cases. Since 2016, LUV were the second most approved drug by the FDA (22%), followed by other types of lipid-based formulations (21%) [25].

In 1995, two liposomal drugs were approved by the FDA, Doxil^®^ and Abelcet^®^, for the treatments of cancer and fungal infections, respectively. Their clinical success contributed to the approval of several new liposomal drugs, mainly as treatments of infections (AmBisome^®^, ArikayceTM), cancer (DaunoXome^®^, Myocet^®^, Mepact^®^, Vyxeos^®^), respiratory distress syndrome in premature infants (Curosurf^®^) and rare genetic diseases (Onpattro^®^), as well as pain management (DepoDur^®^, Exparel^®^) [26,36].

Unfortunately, the emerging antimicrobial drug resistance and the lack of approved antimicrobials have been a public health issue worldwide [37]. On the other hand, EO exhibit intrinsic antimicrobial activity, being widely proposed as a viable alternative to solve this problem [38]. Furthermore, nanotechnology represents a powerful tool for developing new therapeutic strategies to reach different targets efficiently [4]. In this sense, LNP are excellent strategies for EO nanoencapsulation [39], being interesting alternatives to enhancing human health by improving the bioavailability and intrinsic antimicrobial properties of EO [40,41,42,43,44,45], as well as their anticancer [7,20,28,46,47,48], hypolipidemic [49], antioxidant [50], antidiabetic [51], anti-inflammatory and analgesic properties [52,53]. They will exhibit additional relevance once they have biodegradable, biocompatible and ecofriendly properties [5].

### 2.1. Applications to Human Health

Overall, the optimized antimicrobial activity of EO-loaded LNP has been widely described. Exciting results for the treatment of some neglected diseases caused by parasites have been reported. Santos et al. developed chitosan-coated NLCs for *Citrus sinensis* EO delivery against leishmaniasis [54]. Hybrid NLC were composed of a mixture of beeswax and *Citrus sinensis* EO or R-limonene (ratio of 70:30), with concentrations of lipid, surfactant (poloxamer 188) and chitosan of 7, 7 and 0.05%, respectively. The particle sizes ranged from 97.9 to 111.3 nm, and the formulation showed stability for 90 days (25 and 4 °C), which enabled a significant reduction in the viability of promastigote forms of *Leishmania amazonensis* when compared with the free active compounds. Ghanbariasad et al. also investigated the leishmanicidal effect of EO-based lipid formulations [55]. However, they prepared NE composed of *Cinnamomum zeylanicum* EO (0.5%, *v*/*v*) and Tween^®^ 20. The nanoparticle size was around 28.0 ± 4.0 nm and demonstrated physicochemical stability for 6 months when stored at both 4 and 25 °C. After the NE-loading *Cinnamomum zeylanicum* EO treatment, a total reduction in the viability of *L. major* and *L. tropica* promastigotes (~0%) was observed. Moghadam et al. developed optimized NIO formulations that contained *Bunium persicum* EO against the *Trichomonas vaginalis* parasite [41]. This nanosystem was prepared by the thin-layer hydration method using surfactants, cholesterol, distearoyl phosphoethanolamine-polyethylene glycol and *Bunium persicum* EO (1 mg/mL). The encapsulation efficiency (%EE) and particle size were 63.11% and 159.7 nm, respectively, with a suitable stability for 120 days. They observed low nanotoxicity in healthy cells, a higher toxicity in the parasite, and a sustained release profile of the *Bunium persicum* EO at the same temperature and acidity as the *T. vaginalis* microenvironment. Siyadatpanah et al. prepared liposomal systems that contained *Bunium persicum* and *Trachyspermum ammi* EO. The physicochemical properties and in vitro activity against *T. vaginalis* were evaluated [56]. LUV were prepared with phosphatidylcholine and cholesterol using the thin-film evaporation method. The size and zeta potential values of the LUV that contained *B. persicum* were 121.0 nm and −16.7 mV, respectively, whereas for the *T. ammi* EO, such values were 111.0 nm and −9.0 mV, respectively. The %EE of the loaded EO were 51.64 ± 1.24% (*B. persicum* EO) and 40.12 ± 2.71% (*T. ammi* EO). An EO sustained release profile from liposomal systems for 24 h was reported. In addition, a satisfactory parasite growth inhibition when compared with controls was described, demonstrating its potential as a treatment for this type of infection. These findings strongly indicate that EO-based LNP have potential for the treatment of infectious diseases, such as leishmaniasis and trichomoniasis, demonstrating the importance of EO nanoencapsulation, which improves the chemical stability; bioavailability; and, consequently, therapeutic efficacy.

There are many reports of EO nanosystems with activity against bacterial species. Fahimnia and coworkers developed a topical nanohybrid gel that contained lavender EO loaded with SLN and demonstrated in vitro antibacterial activity against *Staphylococcus aureus* [40]. Tween^®^ 80 was employed as the surfactant, lecithin and cholesterol were used as the lipids, and Carbomer 940 was used as the gelling agent. The lavender-EO-based SLN revealed a particle size of 19.24 nm, a PDI of 0.079 and an %EE of 75.46%. It potentiated the antibacterial properties of pure EO due to its sustained release profile, where this property was maintained in the topical gel formulation. Wen et al. developed optimized NLC in gel with activity against *Pseudomonas aeruginosa* [57]. They investigated their topical effect in wound infection. This nanosystem was composed of Labrafac™ lipophilic WL 1349, stearic acid, cinnamon EO (167 mg), Tween^®^ 80 and water, with Poloxamer 407 used as the gelling agent. The EO release test exhibited an initial burst release followed by a sustained release profile. The in vitro and in vivo antimicrobial activities were evaluated by the determination of the minimal inhibitory concentration (MIC) and burnt wound model using male Sprague Dawley rats, respectively. They observed that NLC-based cinnamon EO in gel exhibited significant inhibitory activity against a *P. aeruginosa* strain compared with an NLC-based cinnamon EO formulation and pure cinnamon EO. Furthermore, the treatment with nanohybrid NLC successfully healed wounds in rats in 6 days. In other work, the LUV loading of citral (50 mg/mL) inhibited the proliferation of the *Streptococcus mutans* cariogenic species. The LUV were composed of Lipoid S75 and *Citrus limon* var. *pompia* EO or pure citral [58]. The EO and citral-based LUV exhibited particle sizes that were around 110.0 nm in both cases. The values of the zeta potential were negative, around −85.0 mV (EO-based LUV) and −72.0 mV (citral-based LUV). All the formulations were stable throughout 90 days of storage at 25 °C and demonstrated biocompatibility with epithelial cells, which increased the skin repair rate. However, regarding the antibacterial activity, only citral-loaded LUV provided an inhibition of *S. mutans* proliferation. As suggested by the authors, this formulation could be considered suitable for the development of an effective, safe and pleasant mouthwash for the prophylaxis of oral cavities. Overall, these reports validated the use of EO-based LNP as potential antibacterial agents, improving their bioactivity and shelf time.

LNP can also be applied as a viral infection treatment. Zhang et al. prepared *Artemisia argyi* EO-loaded NLC, investigating its therapeutic effect against the Hepatitis B virus (HBV) in an animal model [42]. The formulation was composed of a mixture of glycerin monostearate with caprylic acid/capric triglyceride, EO (0.1 g), poloxamer 188 and egg yolk lecithin. Such a system showed a particle size of 72.33 ± 1.58 nm, with a PDI of 0.27 and an %EE of 87.49%. The in vivo results show that this nanosystem exhibited a desirable inhibitory effect on the replication HBV DNA of both the serum and liver of ducks, being a potential antiviral agent for the treatment of HBV infection. There is also a report of a hybrid LUV system in gel composed of imiquimod, *Thymus vulgaris* EO and *Oreganum vulgare* EO and functionalized by a DNA aptamer. This system was designed to treat intraepithelial lesions caused by the human papillomavirus (HPV) [59]. The *T. vulgaris* EO (0.25%, *w*/*v*) and *O. vulgare* EO (1%, *w*/*v*) were emulsified by Tween^®^ 80. LUV were produced by the ethanol injection method, using cholesterol, phosphatidylcholine, imiquimod, milli-Q water and EO. Moreover, potassium sorbate, hydroxypropylmethylcellulose and propylene glycol were used as excipients to obtain the gel formulation. The LUV were characterized using Dynamic Light Scattering (DLS), which showed appropriate particle sizes (50.0–200.0 nm) and PDI values ≤ 0.3. The formulations exhibited cytotoxicity in cancer cells without affecting the healthy cells. The results demonstrated that the presence of EO potentiated the imiquimod–LUV in vitro efficacy, while the functionalization increased the selectivity for cervical cancer cells.

EO or their components have also demonstrated an antifungal property [60,61]. Encapsulation in LNP has been explored, with the aim to develop alternative treatments in the face of increasing multidrug resistance. El-Salam et al. developed hybrid liposomal formulations that encapsulated tea tree EO coated by chitosan [62]. The antifungal activity against *Aspergillus flavus*, *Aspergillus fumigatus*, *Microsporum gypsum* and *Fusarium oxysporum* were analyzed through in vitro and in vivo infection models. The hybrid LUV showed a spherical shape, with particles sizes in the range of 360.0–420.0 nm, a monodisperse size distribution (PDI = 0.05) and a zeta potential value of +2.6 mV. The in vitro results demonstrated that these nanosystems show fungicidal activity against the analyzed strains at safe concentrations (≤5 mg/mL), confirming the in vivo biocompatibility and efficacy. An in vivo study on Wister rats revealed that LUV formulations destroyed the fungal cells inside without causing any toxicity to the tissues. Alteriis et al. encapsulated *Lavandula angustifolia* EO in LUV and evaluated its activity against *Candida auris* biofilms [63]. The mean particle size was 177.2 ± 11.5 nm, with PDI < 0.3 and a zeta potential around −3.73 mV. The nanosystem was composed of L-α-phosphatidylcholine, cholesterol and *L. angustifolia* EO (concentration of 10% *v*/*v*), and showed in vitro antifungal and antibiofilm properties against *C. auris*. Baldim et al. evaluated the effect of *Lippia sidoides* EO encapsulation in NLC and their potential efficacy against the *C. auris* multidrug-resistant pathogen [64]. NLC formulations were prepared through the hot emulsification method, followed by sonication. The formulations were based on oleic acid and Compritol^®^ 888, or a combination of carnauba wax and beeswax, and stabilized by sodium dodecyl sulfate. They observed that the lipid matrix composition of NLC affected both the particle size and PDI. At lower surfactant concentrations (1.4 and 2.4% *w*/*w*), the nanocarriers composed of beeswax and carnauba wax presented smaller (301.5 to 318.7 nm) and more homogeneous (PDI 0.19 to 0.22) particles compared with those composed of Compritol^®^ 888 (particles sizes between 321.3 to 445.5 nm and PDI 0.33 to 0.41). However, when a higher concentration of surfactant (4.2%; *w*/*w*) was used, it provided smaller particles (213.1 nm) than the other formulations. In relation to the antifungal efficacy, the NLC demonstrated potent activity against *C. auris* with any toxicity in a *Galleria mellonella* in vivo model. Husein et al. investigated the use of EO (eucalyptus and orange) as a liquid lipid in an NLC preparation [65]. They tested the antifungal activity against *Candida albicans* strains. The formulations were composed of Miglyol^®^ 812 and eucalyptus (35% *w*/*v*) or orange EO (40% *w*/*v*), Kolliphor^®^, distilled water, NaCl and soybean lecithin. Both formulations displayed excellent stability properties over 6 months when stored at 4 ± 1 °C, with particle sizes of 163.1 ± 10.8 for the NLC-based eucalyptus EO and 144.3 ± 1.9 for the NLC-based orange EO. The results indicated that the EO encapsulation into NLC significantly enhanced their antifungal effects. So, these works confirmed the viability of LNP loaded with EO against drug-resistant fungi acting as potential therapeutic strategies.

Considering the antimicrobial and antibiofilm properties of EO and the previously reported success of their encapsulation in LNP, a complementary approach was explored for the preparation of sanitizers for healthcare applications. Gross et al. developed novel sanitizer tablets that contained clove-EO-loaded NE [66]. These tablets presented antimicrobial and acaricide activities, and were prepared by the wet granulation of spray-dried soap. A blend of nonylphenol ethoxylate and coconut fatty acid diethanolamide surfactants, water and clove EO (concentration of 12.5%) was employed. The structural characterization revealed a particle size of 100.0 nm, PDI of 0.3 and zeta potential of −19.5 mV. Then, such formulations were incorporated into soap solid matrices. These new systems presented a particle size close to 221.0 nm, PDI of 0.5 and zeta potential of −17.2 mV. Antimicrobial activity assays were carried out against Gram-negative (*Escherichia coli*) and Gram-positive (*Staphylococcus aureus*) bacterial strains and yeast (*Candida krusei*). The acaricidal and larvicidal activities were analyzed against ticks (*Rhipicephalus microplus*). The results demonstrated the efficacy of the sanitizer tablet against bacteria, fungi, mites and larvae. Such systems can be used both in healthcare for effective surface disinfection and in animal livestock to control pests that affect animal health. Drais et al. developed a hybrid NE gel skin sanitizer and evaluated the antibacterial activity against *Staphylococcus aureus* and *Escherichia coli* [67]. The formulations were composed of peppermint and myrtle EO (10% *w*/*w*; in a 1:1 ratio), Tween^®^ 80 and propylene glycol, using carbomer 940 as the gelling agent. The mean particle size, PDI and zeta potential values ranged from 25.83 to 55.86 nm, 0.26 to 0.38 and −12.6 to −19.6 mV, respectively. In vitro antimicrobial activities against *S. aureus* and *E. coli* were presented. The authors claimed the nanosystems as alternatives in the process of cleansing the skin against these microorganisms. Mechmechani et al. reported that the encapsulation of functional enzymes combined with EO acted as a promising anti-biofilm active that was able to be applied in the food and medical fields [68]. Considering these reports and the constant trend toward green biotechnology, there are several insights to be explored in the development of antiseptic/disinfectant nanoformulations.

These results support the fact that EO nanoencapsulation by different LNP can be innovative therapeutic agents for the treatment of infections caused by viruses, parasites, bacteria and fungi. Their application as sanitizers is also an interesting alternative to be explored more. Furthermore, many studies validated the use of LNP as potential nanosystems for the delivery of lipophilic active molecules, improving the physicochemical stability and reducing their volatility and toxicity [5].

### 2.2. Applications in Veterinary Medicine

The application of nanotechnology is valuable in a wide range of fields of knowledge, including in animal health. The benefits of encapsulating antimicrobial agents as a viable alternative to the use of antibiotics have already been discussed [69]. Such uses extend to the area of nutrition. Once it can improve animal digestion, it is able to increase the absorption of nutrients, resulting in a higher quality of products intended for human feeding, such as meat, milk and eggs [70,71]. In fact, LNP application is still limited in animal production and the prevention and treatment of diseases [69]. Moreover, LNP should be applied to all groups of animals, from companion to farming animals, which is clarified throughout this review.

Broilers were investigated in order to achieve a high performance in feed conversion to ensure rapid growth. However, they are easily affected by bacterial diseases caused by failures in their immune system. Meligy et al. encapsulated oregano (150 mg), cinnamon (200 mg) and clove (50 mg) EO by LUV. Then, they were added to the animal diet to understand their antioxidant and antimicrobial properties, as well as the changes in the intestinal microbiota of broilers from 1 to 38 days [72]. A decrease in intestinal bacterial species, such as *Clostridium*, *Escherichia* and *Enterobacteriaceae*, were observed when supplementation (300 and 400 mg/kg) with LUV occurred, as quantified by real-time PCR. Pires et al. developed different NLC formulations that contained several EO (geranium, lemongrass, clove, cinnamon and oregano) [19]. These NLC showed in vitro antimicrobial activity against *Campylobacter* isolated from chicken carcasses, with lemongrass, cinnamon and geranium EO being the best bioactives. The NLC showed sizes of 151.8–179.2 nm, 245.4–231.8 nm and 208.4–219.7 nm, respectively; PDI values from 0.13 to 0.23; and zeta potentials around −28.8 to −40.0 mV. All the systems were stable for 210 days.

The market for pets has exponentially grown over the years, where the constant searching for higher longevity and quality of life means looking for more efficient and convenient therapies. In this sense, NLC loaded with clove EO (19.75%) and α-mangostin (0.25%) were developed as bioactives, with Span 80 (2–4%) as the surfactant, with the aim to control canine periodontitis since low bioavailability is currently observed with the commercially available forms. Satisfactory results were observed in the penetration tests in gingival tissues. The three-dimensional confocal light scanning microscopy indicated that the nanostructured compound resulted in better penetration into the gingival epithelium, being also able to inhibit the growth of *E. coli*, *Salmonella* spp. and *Klebsiella pneumoniae* isolated from the oral cavities of dogs. The NLC had a particle size less than 200.0 nm, PDI values between 0.1 and 0.2 and a zeta potential up to −30 mV. The formulations were stable for 60 days when stored at room temperature [33]. This field should be more widely explored.

The development of NE based on tea tree EO with antifungal activity against the *Pythium insidosum*, which causes pythiosis, which affects humans and other animals (mainly horses), was reported. NE with 1% *M. alternifolia* EO showed sizes smaller than 200 nm, a PDI below 0.25 and a negative zeta potential. NE-based EO presented the highest capacity to inhibit *Pythium insidosum* (around 90%) in vitro [73].

The use of nanotechnology in livestock farming can be diverse, including in diagnosis, therapeutics, vaccines, nutraceuticals and thermal insulation [74]. Unfortunately, the applications of LNP loaded with EO with antimicrobial, antifungal and antiparasitic activity for veterinary clinical uses are still scarce. Nevertheless, the abovementioned reports make it clear that such innovations demonstrated robust evidence in the development of biodegradable and easily scalable nanoproducts based on green chemistry.

### 2.3. Applications as Biopesticides/Biorepellents

The global agricultural sector faces major challenges due to plant/animal diseases caused by pests, such as bacteria, fungi and insects. In this context, plant-based nanopesticides have emerged as promising solutions that incorporate substances on the nanometric scale. LNP in particular stand out for their ability to release active compounds in a sustained manner, which increase their efficacy and decrease their toxicity [75].

Pesticides are chemical compounds that are derived from natural or synthetic sources and are designed to control pests and insects [76]. On the other hand, repellents are products that prevent diseases transmitted by mosquitoes and ticks, as well as repel or mitigate mosquitoes and pests [77].

There are a range of diseases that disturb the productivity of farm animals and severely affect meat production. Urban pests have also posed threats to human health and well-being. Chemical pesticides remain the most used active for prevention [78]. However, these chemicals are often associated with undesirable environmental impacts, such as soil and groundwater contamination, and causing toxic effects on other organisms, such as pollinators [79]. Thus, LNP have emerged as alternative nanosystems for pest management. LNP associated with EO have been employed in several approaches, including as larvicidal/adulticidal [8,80,81], acaricidal [82], insecticidal [34,83] and antimicrobial [19].

Overall, some works demonstrated that LNP encapsulating EO exhibit excellent pesticidal activity. Radwan et al. developed NLC based on stearic acid, green tea and fennel EOs [8]. The NLC had particle sizes between 277.0 and 287.0 nm, PDI values below 0.1 and zeta potential values between −28.22 and −34.31 mV. They showed larvicidal and adulticidal activities against *Cullex pipiens*. Hikal et al. formulated NE using oleic acid, sodium glycocholate, and polysorbate 20 honeysuckle or patchouli EO delivery. These NE were tested against the larvae and adults of *Culex pipiens*, where they showed particle sizes around 183.9–250.2 nm, PDI values in the range from 0.285 to 0.420 and zeta potentials of −49.8 and −23.5 mV. The data revealed that the honeysuckle-EO-based NE and patchouli-EO-based NE were more effective than the respective pure EO against *C. pipiens* larvae and adults [80].

Rodrigues et al. developed and characterized NE that contained *Ayapana triplinervis* EO to test the larvicidal activity against *Aedes aegypti* larvae, the vector of the Dengue virus [81]. These nanosystems showed the expected activity, with particle size averages between 88.3 and 99.5 nm, PDI values below 0.2 and zeta potentials ranging from −17.7 to −32.8 mV. Dunan et al. described EO-based NE and evaluated their entomotoxicity and phytotoxicity properties against *Bemisia tabaci*, which affects tomatoes [84]. The NE were composed of Tween^®^ 80, ethanol, and artemisia EO or rosemary EO (1%). The structural characterization was carried out for freshly prepared formulations and no long-term stability study was carried out. Particle sizes of 217.0 and 199.0 nm, PDIs of 0.36 and 0.42, and zeta potentials of −18.63 and 12.27 mV were obtained for the NE prepared with the rosemary EO and artemisia EO, respectively. The entomotoxic and phytotoxic bioassays were performed in climatic chambers by fumigation. An artemisia-EO-based NE and rosemary-EO-based NE resulted in *B. tabaci* mortalities of 60% and 98%, respectively, being the most efficient tested treatments. In terms of phytotoxicity, the highest index was found by a rosemary-EO-based NE. Furthermore, Manjesh et al. reported on NE based on *Pogostemon cablin* EO (obtained from leaves) using a biosurfactant (saponin). Its insecticidal efficacy was investigated against *Tetranychus urticae* adults and *Spodoptera litura* larvae [85]. The stability of the prepared NE was measured at room temperature and under an accelerated storage condition (54 ± 1 °C) for 14 days. The particle sizes ranged from 46.2 to 71.7 nm with PDIs of 0.49 to 0.69 and zeta potentials of −22.62 to −29.21 mV. The NE displayed the highest efficacy against *T. urticae*, where they exhibited considerable antifeedant action against *S. litura* larvae.

Mites are also pests that hinder animal welfare. Hoda et al. developed and tested the toxicity of NE composed of polyethylene glycol, Tween^®^ 80, and *Artemisia herba-alba* or *Melia azedarach* EO [82]. Transmission electron microscopy analysis revealed particle sizes of 52–91.0 nm for the NE loaded with the *M. azedarach* EO and 62–69.0 nm for those loaded with the *A. herba-alba* EO. Both formulations showed in vivo acaricidal activity against *H. dromedarii*. Teng et al. proposed NE against the house dust mite *Dermatophagoides farinae* through fumigant, repellent and larvicidal assays. An 80% mortality rate of larvae treated with NE was observed, and thus, they acted as an excellent larvicide. When compared with the pure EO, the NE showed suitable acaricidal activity after 48 h of treatment, but poor fumigant effects against adult *D. farinae* at 12 and 24 h. The EO-based NE repellent effect against *D. farinae* was found to be stable over time [86]. Barbhuiya et al. developed NIO loaded with an *Azadirachta indica* EO and evaluated the in vitro antimicrobial activity against two pathogens that affect the plants *Xanthomonas* and *Pantoea*. These systems were composed of neem EO, Tween^®^ 80 and soy lecithin. The nanoformulation showed a particle size of 93.3 nm, and when under refrigeration between 4 and 8 °C, showed an increase in size after 30 days of storage. There was a significant increase in the antimicrobial inhibition growth against both bacteria, with 10 mg/mL of neem-EO-loaded NIO [87]. Adamu et al. reported a ginger-EO-based NE and evaluated its potential to manage the bacterial disease of rice caused by *Xanthomonas oryzae*. The NE was composed of Tween^®^ 20 and ginger EO, and it showed a particle size of 73.0 ± 0.8 nm, zeta potential of −43.2 mV and PDI of 0.21. The antimicrobial activity of the developed nanoformulation was evaluated by the disk diffusion test and a glasshouse trial experiment. The NE efficacy assay showed a concentration-dependent profile, where 125 μL/mL was the most effective concentration in the control, as well as bacterium growth reduction and disease severity index suppression in rice. In addition, the treatment with a ginger-EO-based NE had no phytotoxicity effects, where it displayed the best performance in the reproductive, grain filling and ripening stages, and improved the rice yield [88].

Other insect species are also targets for urban and agricultural control. Palermo et al. proposed some nanosystems against *Tribolium confusum.* They used several EO that were loaded by NE and nanogels. All formulations showed particle sizes between 95.0 and 144.3 nm and PDI values from 0.14 to 0.25. The in vitro repellent activity and acute toxicity assays were carried out by cold aerosol assays. The best nanoformulation was based on anise EO due to its low toxicity [83]. Múnera-Echeverri et al. developed NLC loaded by thyme and rosemary EO for the targeting of common pests, such as *M. persicae*, *T. urticae* and *F. occidentalis*, which affect ornamental flowers. The compositions of these NLC included carnauba wax, Tween^®^ 80 and Mygliol, and EO as liquid lipids. It was observed that the NLC production method affected the physicochemical parameters. The high shear homogenization method was selected as the best technique to prepare the NLC. The thyme EO-NLC and rosemary EO-NLC had particle sizes of 347.8 and 288.1 nm, a PDI of 0.18 (both cases), zeta potentials of −33.8 and −34.0 mV, and %EE of 71.9 and 80.6%, respectively. The assessment of contact toxicity on leaf disks showed that the NLC that carried the thyme EO reached mortality rates between 24 and 38% against *F. occidentalis*, and thus, demonstrated moderate insecticidal activity. On the other hand, a rosemary-EO-based NLC showed efficacy against all the pests [34]. Tortorici et al. described NLC loaded with rosemary, lavender or peppermint EO. They were tested against *Aphis gossypii*, *Spodoptera littoralis* and *Tuta absoluta*. The formulations were prepared using Softisan^®^ 100 as a solid lipid and a surfactant mixture composed of Kolliphor^®^ RH40 and Labrafil^®^, and loading the EO with a concentration of 10% *w*/*v*. The results demonstrated a stable colloidal formulation characterized by a homogeneous particle distribution (mean particle size <200.0 nm and PDI < 0.3), with a high amount of EO encapsulated (≥70%). The rosemary-EO-based NLC was the most promising system since it exhibited excellent stability. All the NLC were able to induce a high mortality and reduce the *A. gossypii* progeny significantly by topical contact exposure. However, the lavender- and rosemary-EO-based NLC were able to decrease the feeding activity of *S. littoralis* [89].

It is clear that the application of nanobiotechnology processing EO as nanoformulations constitutes a broad field of research that allows for different approaches to control urban and agricultural pests while reducing the environmental and human health impacts. Table 1 summarizes the available data with applications in the human, veterinary and environmental fields.

## 3. The Role of LNPs in Industrial Development

The industrial potential of LNP-based pharmaceutical products has been increasingly recognized. It has been stimulated by the urgency of novel efficient and safe drugs against microbials. However, the approval of LNP is still a challenge, particularly regarding the scalability, sterilization and regulatory compliance [14,101,102]. Overcoming these challenges is essential for the successful industrial development, commercialization and clinical translation of LNP. In this sense, the use of Quality-by-Design (QbD) approaches [101], machine learning [103] and process analytical tools, combined with advanced production techniques [104], means it is possible for LNP to reach the market in the near future. Using natural products brings additional challenges to standardizing the chemical characterization of nanoformulations. Some factors, such as the geographic origin, climate, extraction methods and harvest time, can affect the chemical composition and quality of an EO [105]. In addition, green extraction techniques should be promoted for the large-scale harvesting of natural products. This will help to preserve biodiversity by focusing on using sustainable practices without compromising the extraction yields. Innovative technologies, such as ultrasound, microwave, or subcritical and supercritical fluid extraction must be employed [106]. Furthermore, continuous efforts to harmonize regulatory issues for the clinical trials of LNP at a global level are crucial to allow for significant industrial production [101].

## 4. Conclusions

Lipid-nanoparticle-based essential oils represent an innovative approach with high potential to be explored for the development of alternative antimicrobial formulations. Such systems are excellent for the encapsulation of EO, protecting them from light, oxidation and hydrolysis, thus preserving their bioactivity. The analyzed reports confirmed that these nanosystems can be explored in a wide field of applications. They can be used in human health as antimicrobial treatments, topical disinfectants, sanitizers or antiseptics; in animal health as an alternative use of synthetic antibiotics, antifungals and antivirals; and in agriculture to control pests that affect different types of plants. However, more research is still needed to evaluate the shelf time and safety of these nanosystems, as well as their potential impacts on the environment.

## 5. Perspectives

In the last few years, the world has been facing different health emergencies, which resulted in the COVID-19 pandemic together with different drug multiresistant microbial species, which has caused millions of deaths and public spending on hospitalizations and ICUs. Many efforts have been focused on the search for new antimicrobials candidates that can be effective against such mutant microbial species. However, the deaths related to generalized infections and sepsis have been exponentially growing around the world.

The ancient science is robust in demonstrating the effectiveness of diverse plant species against different pathogens that affect human, environment and animal health. Moreover, it was clear that processing such biological compounds in nanostructured systems improved their physicochemical, pharmacokinetics and pharmacodynamics properties, which resulted in formulations that can be administered using several routes or clinical and industrial surfaces.

Considering the urgency of the development of new drugs, the lack of specific regulation for the approval of proven safe and effective nanodrugs is the major challenge to be overcome around the world. Specifically in the case of LPN, the commonly used excipients are GRAS and those widely employed by food and cosmetic industries: such systems are biodegradable, do not accumulate in visceral organs such that they cause systemic toxicity; the storage, transport and distribution do not require special conditions; and they are easily scaled up with a long-term shelf life, which results in low-cost industrial production. In order to advance the industrial developments of such forms, the triple helix approach should be explored to combine multidisciplinary efforts of the universities, regulatory agencies and industries to provide standardization of the production, evaluation and waste disposal methods for scalable nanostructured drugs. The recognition of nanoformulations as commercially safe and effective drugs is important.

## Figures and Tables

**Table 1:** Lipid nanoparticles loaded with different essential oils with antimicrobial activity and their uses in the human/animal health and environmental fields.

LNP	System Composition	EO Loaded	EE (%)	Stability Data			Target	Biological Tests	Results	Reference
				Size (nm)	Storage Temperature	Storage Time (days)				
Human Applications										
LUV	1,2-distearoyl-sn-glycero-3-phosphocholine, l-α-phosphatidylcholine from egg yolk, curcumin and EO	*Lippia * *origanoides*	-	110.0–115.6	4 °C	60	*Staphylococcus aureus*	In vitro antimicrobial activity and assessment of cytotoxic activity in vitro.	LUV composed by L-α-phosphatidylcholine and 600 μg/mL of EO and curcumin inhibited bacterial growth by approximately 65%, which showed statistically significant differences compared with the control. No cytotoxicity toward VERO cells was observed.	[37]
Multiple lipid nanoparticles	Glyceryl trioleate, glyceryl tristearate, Pluronic^®^ L64, Tween^®^ 80 and EO	Rosemary	31.0–51.0	~110.0	4 °C	60	*Pseudomonas aeruginosa*	Antimicrobial susceptibility test by MIC.	Considerable increase in the antibacterial property of EO-loaded lipid nanoparticles.	[38]
SLN gel	Tween^®^ 80, soya lecithin, cholesterol, dichloromethane, Carbomer® 940, EO, propylene glycol and triethanolamine	Lavender	75.4	<20.0	25 °C	-	*Staphylococcus aureus*	Antibacterial assay (MIC, MBC, disk diffusion method) and cell compatibility assay.	The SLN potentiated the antibacterial properties of the EO. The incorporation of SLN in the gel matrix enhanced the sustained delivery of the EO. No toxic effects were observed.	[40]
NIO	Tween^®^ 80, cholesterol, distearoyl phosphoethanolamine-polyethylene glycol, EO	*Bunium persicum*	63.1	159.7	4 °C	120	*Trichomonas vaginalis*	Cytotoxicity tests with human foreskin fibroblasts cell line and in vitro activity against *T. vaginalis*.	Low cytotoxicity of NIO on healthy cells and high toxicity on *T. vaginalis* parasite.	[41]
LNP	System Composition	EO Loaded	EE (%)	Stability Data			Target	Biological Tests	Results	Reference
				Size (nm)	Storage Temperature	Storage Time (days)				
Human Applications										
NLC	Glycerin monostearate, caprylic acid/capric triglyceride, poloxamer 188, egg yolk lecithin and EO	*Artemisia argyi*	87.5	72.3	4 °C	-	Hepatitis B virus	Anti-HBV activity in vitro and in vivo and acute toxicity in vivo.	EO-loaded NLC displayed potent in vitro and in vivo anti-HBV activity with improved oral bioavailability and liver exposure.	[42]
NLC	Ucuuba butter, poloxamer 188 and essential oil	*Cynnamomum zeylanicum*, *Origanum vulgare*, *Melaleuca artenifolia* and *Mentha pipperita*	-	<300	25 °C, with thermal cycling stability at 40 °C and 15 °C	365	*A. baumannii*, *P. aeruginosa, K. pneumoniae*	In vitro antibacterial activity (MIC) and in vivo nanotoxicity assay on a chicken embryo model.	NLC demonstrated long-term stability and showed antibacterial activity. The cinnamon-EO-based NLC was the most efficient system at inhibiting bacterial growth, and was safe under all conditions tested in nanotoxicity test.	[43]
NLC	Beeswax, Poloxamer 188, chitosan and *Citrus sinensi* OE	*Citrus sinensi*	-	103.6	4 and 25 °C	90	*Leishmania infantum* and *Leishmania amazonensis*	Leishmanicidal activity and in vitro cytotoxicity in L929 and RAW 264.7 cells.	NLC significantly reduced the viability of promastigote forms of *L. amazonensis*. Chitosan-coated NLC provided better antileishmanial activity than uncoated NLC.	[54]
NE	Tween^®^ 20 and cinnamon EO (0.5%)	*Cinnamomum zeylanicum*	-	52.0	4 and 25 °C	180	*Leishmania major* and *Leishmania tropica*	Leishmanicidal activity assay in vitro.	NE improved the anti-leishmania activity.	[55]
LNP	System Composition	EO Loaded	EE (%)	Stability Data			Target	Biological Tests	Results	Reference
				Size (nm)	Storage Temperature	Storage Time (days)				
Human Applications										
LUV	Soybean phosphatidylcholine, cholesterol and EO	*Bunium persicum* and *Trachyspermum ammi*	51.64 and 40.12	≤121.0	-	-	*Trichomonas vaginalis*	Cytotoxicity studies (MTT assay), cellular uptake study and determination of IC_50_ LUV on *T. vaginalis*.	Cellular studies showed that the LUV had a low toxicity for healthy cells but showed toxicity to *T. vaginalis.* The LUV were well absorbed by the cells. The IC_50_ values of the LUV composed of *B. persicum* and *T. ammi* were calculated to be 14.41 and 8.08 μg/mL in *T. vaginalis* after 24 h, respectively.	[56]
NLC gel	Tween^®^ 80, Poloxamer 407, Labrafac™ lipophile WL 1349, stearic acid and EO	*Cinnamomum zeylanicum*	95.4	108.5	25 °C	-	*Pseudomonas aeruginosa*	Determination of MIC, in vivo burnt wound study and histological examination.	Gel containing NLC effectively entrapped the cinnamon EO, significantly improved wound healing and exhibited an antimicrobial effect.	[57]
LUV hybrid	Cholesterol, phospholipid, chitosan and *tea tree* EO	*Tea tree*	-	360.0–420.0	−20 °C	-	*Aspergillus flavus*, *Aspergillus fumigatus*, *Fusarium oxysporum* and *Microsporum gypsum*	Antifungal in vitro assay (MIC, MFC), cytotoxicity study and in vivo antifungal activity analyses in a rat model.	Hybrid LUV showed MIC and MFC values that ranged between 0.039 and 1.25 and 0.0195 and 0.625 mg/mL, respectively. A study of the cytotoxicity on liver cells recorded safety levels at a concentration of 5 mg/mL. In vivo assays revealed formulation antifungal activity without causing any noticeable toxicity.	[62]
LNP	System Composition	EO Loaded	EE (%)	Stability Data			Target	Biological Tests	Results	Reference
				Size (nm)	Storage Temperature	Storage Time (days)				
Human Applications										
LUV	l-α-phosphatidylcholine, cholesterol and *L. angustifolia* EO	*Lavandula angustifolia*	100.0	177.2	-	-	*Candida auris*	Antifungal activity on planktonic cells and biofilms of *C. auris.*	Significative growth reduction was observed on planktonic fungi when the cells were incubated in the presence of LUV in an EO concentration of 0.01% *v*/*v*. The eradication of biofilms was obtained at higher concentrations of EO (0.5% *v*/*v*).	[63]
NLC	Compritol^®^ 888 ATO, Carnauba wax and Beeswax, oleic acid, sodium dodecyl sulfate and *L. sidoides* EO (1.8 % *w*/*w)*	*Lippia sidoides*	90–100	213.1–445.5	25 °C	-	*Candida auris*	Antifungal activity (MIC and MFC) and in vivo toxicity assay.	*Candida auris* was inhibited by both the EO and EO-loaded NLC with concentrations between 0.281 and 0.563 mg/mL. The highest anti-*Candida* activity was achieved with the NLC containing higher SDS concentrations in the composition. No toxicity was observed at the MIC concentration.	[64]
NLC	Miglyol^®^812, Kolliphor^®^ HS 15, NaCl, soybean lethicin, and eucalyptus EO (35% *w*/*v*) or orange EO (40% *w*/*v*)	Eucalyptus and orange	-	<165.0	4 °C	180	*Candida * *albicans*	In vitro antimycotic activity.	Excellent antifungal activity by NLC loading both EO.	[65]
LNP	System Composition	EO Loaded	EE (%)	Stability Data			Target	Biological Tests	Results	Reference
				Size (nm)	Storage Temperature	Storage Time (days)				
Human Applications										
NLC	Precirol^®^, oleic acid, poloxamer^®^ 188 or cetyltrimethylammonium bromide, and *Lippia sidoides* EO (3% *w*/*w*) or *Syzygium aromaticum* EO (3% *w*/*w*)	*Lippia sidoides* and *Syzygium* *aromaticum*	84.6–100.0	<161.0	25 °C, with thermal stress at 37 °C and 5 °C	28	SARS-CoV-2	Virucidal activity against SARS-CoV-2.	NLC that contained a quaternary ammonium surfactant and a mixture of *L. sidoides* EO and *S. aromaticum* EO (1:1) showed enhanced virucidal activity compared with formulations that contained a pure EO.	[90]
NE	Tween^®^ 80 and clove EO	*Syzygium aromaticum*	-	32.67	25 °C	40	*B. cereus*, *S. aureus*, *E. coli*, *K. oxytoca*, *C. albicans*, *C. neoformans*, *A. brasiliensis*, *A. flavus* and *A. fumigatus*	In vitro antibacterial activity (MIC and antibiofilm activity study) and antifungal activity (MIC and MFC).	NE showed antibiofilm and antifungal activity significantly higher than control. NE exhibited MIC values against *B. cereus*, *S. aureus*, *E. coli* and *K. oxytoca* that ranged from 0.31 to 5 mg/mL and MBC between 0.62 and 10 mg/mL. For fungus strains, the MICs ranged from 0.78 to 12.5 mg/mL and the MFCs from 1.56 to 12.5 mg/mL.	[91]
LUV	Lecithin, cholesterol, dichloromethane/methanol and *Zhumeria majdae* EO	*Zhumeria majdae*	92.3	118.0	4° C	-	*A. baumannii*, *P. aeruginosa*, *S. typhimurium*, *K. pneumoniae*, *S. aureus*, *E. faecalis*, *E. coli, S. bovis*	Anti-biofilm activity, anti-quorum sensing activity, disk diffusion method, MIC, MBC, antioxidant activity and cytotoxicity study.	LUV had better antioxidant, anti-quorum sensing, and anti-biofilm activities than the free EO, along with biocompatibility.	[92]
LNP	System Composition	EO Loaded	EE (%)	Stability Data			Target	Biological Tests	Results	Reference
				Size (nm)	Storage Temperature	Storage Time (days)				
Human Applications										
NLC hybrid	Tween^®^ 80, xanthan gum, Precirol^®^ and *Carum carvi* EO	*Carum carvi*	94.1	40.0–250.0	25 °C	-	*Escherichia coli*, *Staphylococcus aureus*, *Staphylococcus epidermidis* and *Pseudomonas aeruginosa*	In vitro antibacterial activity (disc diffusion, MIC and MBC) and infected wound-healing in vivo assay.	The results show better antibacterial activity of the EO-based NLC in comparison with pure EO. Topical application of the EO-NLC decreased the bacterial colonization, shortened the inflammatory phase and promoted the proliferative phase in mice cutaneous wounds.	[93]
LUV	Soybean lecithin, cholesterol, chloroform, EO, Tween^®^ 80, phosphate-buffered saline solution	*Origanum vulgare*	79.5	<111.0	4 °C	30	*Trichophyton rubrum*	In vitro antifungal activity: mycelial growth test.	Encapsulation of the EO in nanoliposomes was effective at increasing the antifungal activity against *T. rubrum* strains.	[94]
LUV	Lipoid S75, mixture of soybean phospholipids and EO.	*Citrus limon*	~86.0	152.0	25 °C	-	*E. coli*, *P. aeruginosa*, *S. aureus* and *Candida albicans*	In vitro antimicrobial tests (agar diffusion method and micro-dilution method) and in vitro biocompatibility.	The antimicrobial activity of the LUV was confirmed against all pathogens tested, and they demonstrated high biocompatibility in human keratinocytes.	[95]
NLC	Miglyol®, Precirol®, Poloxamer and *Mentha pulegium* EO	*Mentha* *pulegium*	94.2	40.0–250.0	25 °C	-	*S. pneumoniae*, *S. epidermidis*, *S. aureus*, *P. aeruginosa*, *E. coli*, *L. monocytogenes*, *B. anthracis* and *S. typhimurium*	In vitro antibacterial activity (MIC and MBC), wound-healing assay and histopathological analyses.	Topical application of the EO-NLC reduced bacterial count under in vitro and in vivo assays, which favored wound healing.	[96]
LNP	System Composition	EO Loaded	EE (%)	Stability Data			Target	Biological Tests	Results	Reference
				Size (nm)	Storage Temperature	Storage Time (days)				
Human Applications										
Hybrid NE	Tween^®^ 80, chitosan, sodium alginate and thyme EO (1%)	Thyme	70.0	<100.0	25 °C	-	*E. coli* and *S. aureus*	In vitro antibacterial test and cytotoxicity test.	Antibacterial activity by limiting the growth of Gram-negative and Gram-positive bacteria and showed lower toxicity than the controls.	[97]
NLC gel	Miglyo^®^ 812, Precirol^®^ATO 5, Poloxamer^®^, xanthan gum and *Peppermint* EO	*Peppermint*	93.2	<250.0	-	-	*E. coli*, *S. typhimurium*, *P. aeruginosa*, *S. aureus*, *S. epidermidis*, *B. antracis*, *S. pneumonie, L. monocytogenes*	In vitro antibacterial activity (MIC and MBC) and in vivo infected wound healing in mice model.	Antibacterial activity against Gram-positive and Gram-negative bacteria. The healing process of the infected wound was accelerated, with a decreased bacterial count and edema score.	[98]
NLC	Kolliphor^®^ RH40, Labrafil^®^, Softisan^®^ 100, Tween^®^ 80, clotrimazole, and *Lavandula* EO or *Rosmarinus* EO	*Lavandula* and *Rosmarinus*	-	<200.0	25 °C	180	*Candida* sps.	In vitro antifungal susceptibility test.	Antifungal activities of both EO were retained when they were loaded into the NLC formulations, and the co-existence of clotrimazole and EO led to an improvement in the antifungal effect.	[99]
NE	Tween 80, sandalwood EO and Ultrez	*Santalum album*	-	<230.0	25 °C	120	*B. subtilis*, *S. aureus*, *E. coli* and *P. aeruginosa*	In vitro antibacterial activity (MIC, MBC, agar diffusion assay, trypan blue assay).	EO-based NE showed activity against all bacteria analyzed, with greater antibacterial effects against *E. coli*, *S. aureus* and *P. aeruginosa.* These NE were stable for 2 months.	[100]
LNP	System Composition	EO Loaded	EE (%)	Stability Data			Target	Biological Tests	Results	Reference
				Size (nm)	Storage Temperature	Storage Time (days)				
Animal Applications										
NLC	Cocoa butter, beeswax, murumuru butter, poloxamer® solution and EO	Geranium, lemongrass, clove, cinnamon and oregano	-	151.8–245.4	25 °C	210	*Campylobacter spp*	MIC	They were able to inhibit bacterial growth at a low concentration (0.2 mg/mL).	[19]
NLC	Spam 80, clove EO with α-mangostine	Clove	-	<200.0	25 °C	60	*E. coli*, *Salmonella* spp. and *Klebsiella pneumoniae*	Penetration tests on gingival epithelial tissue.	The NLC had better penetration into the gingival epithelium, in addition to inhibiting the bacterial growth in the oral cavities of dogs.	[33]
LUV	Oregano, cinnamon and clove EO	Oregano, clove and cinnamon	-	-	-	-	*Clostridium*, *Escherichia* and *Enterobacteriaceae*	Real-time PCR.	A decrease in intestinal bacterial populations was observed when broiler diets were supplemented with 300 and 400 mg/kg of LUV.	[72]
NE	*M. alternifolia* EO	*M. alternifolia*	-	<200.0	-	-	*Pythium insidosum*	MIC	There was positive antifungal activity, with the capacity to inhibit around 90% of the growth.	[73]
Agricultural Applications										
NLC	Stearic acid and green tea	Fennel	-	277.0–287.0	-	-	*Cullex pipiens*	Adulticidal/larvicidal assays.	The results show the nanosystem’s potential larvicidal and adulticidal activity.	[8]
NLC	Carnauba wax, Tween® 80 and Mygliol	Rosemary and thyme	-	288.1 and 347.8	-	-	*M. persicae*, *T. urticae* and *F. occidentalis*	Assessment of contact toxicity on leaf discs.	The rosemary-EO-based NLC showed efficacy against all tested pests and the thyme EO-NLC, moderate insecticidal activity.	[34]
LNP	System Composition	EO Loaded	EE (%)	Stability Data			Target	Biological Tests	Results	Reference
				Size (nm)	Storage Temperature	Storage Time (days)				
Agricultural Applications										
NE	Oleic acid, sodium glycocholate and polysorbate 20	Honeysuckle and patchouli	-	183.0–250.2	-	-	*Cullex pipiens*	Adulticidal/larvicidal assays.	The NLC-EO managed to reduce the larvae and had an adulticidal role, which was more effective than the respective EO.	[80]
NE	Sorbitan monooleate and polyoxyethylene sorbitan monooleate	*Ayapana triplinervis*	-	88.3–99.5	-	-	*Aedes aegypti*	Larvicidal/insecticidal assays.	The system increased the mortality rate.	[81]
NE	Polyethylene glycol and Tween® 80	*Artemisia herba-alba* and *Melia azedarach*	-	<100.0	-	-	*Hyalomma dromedarii*	Acaricidal assay.	Both EOs showed acaricidal activity in vivo.	[82]
NE	Tween® 80 and ethanol	*Artemisia* and *Rosemary*	-	199.0–217.0	-	-	*Bemisia tabaci*	Entomotoxic and phytotoxic bioassays.	Artemisia-EO-based NE and rosemary-EO-based NE resulted in *B. tabaci* mortalities of 60% and 98%, respectively.	[84]
NE	Saponin	Pogostemon cablin	-	46.2–71.7	54 ± 1 °C	14	*Tetranychus urticae* and larvae of *Spodoptera litura*	Insecticidal assay.	NE formulations showed a high efficacy against *T. urticae* and antifeedant action against *S. litura* larvae.	[85]
NE	Tween 80, deionized water and ethanol	*Moutan Cortex*	-	150.0	-	-	*Dermatophagoides farinae*	Fumigant, repellent and larvicidal assays.	EO-based NE showed good acaricidal activity at 48 h but poor fumigant effects against adult *D. farinae* at 12 and 24 h.	[86]
NIO	Tween® 80 and soy lecithin	*Azadirachta indica*	-	93.31	4–8 °C	30	*Xanthomonas* and *Pantoea*	In vitro antimicrobial activity.	A significant increase in antimicrobial inhibition, with maximum inhibition at 10 mg/mL of EO-NIO.	[87]
LNP	System Composition	EO Loaded	EE (%)	Stability Data			Target	Biological Tests	Results	Reference
				Size (nm)	Storage Temperature	Storage Time (days)				
Agricultural Applications										
NE	Tween® 20	Ginger	-	73.0	-	-	*Xanthomonas oryzae* pv. *oryzae*	Disc diffusion method and glasshouse trial experiment.	The NEs efficacy was concentration dependent, where it reduced the bacterium growth in the rice plant leaf and suppressed the disease severity index.	[88]
NLC	Softisan^®^ 100, Kolliphor^®^ RH40 and Labrafil^®^	Rosemary, lavender and peppermint	-	<200.0	-	-	*Aphis gossypii*, *Spodoptera littoralis* and *Tuta absoluta*	Antifeedant assay.	The rosemary-EO-based NLC was the most promisor system, where it exhibited excellent stability.	[89]

## Data Availability

Not applicable.

## References

[1] Katopodi A., Detsi A. (2021). Solid Lipid Nanoparticles and Nanostructured Lipid Carriers of natural products as promising systems for their bioactivity enhancement: The case of essential oils and flavonoids. Colloids Surf. A Physicochem. Eng. Asp..

[2] Weisany W., Yousefi S., Tahir N.A.-R., Golestanehzadeh N., McClements D.J., Adhikari B., Ghasemlou M. (2022). Targeted delivery and controlled released of essential oils using nanoencapsulation: A review. Adv. Colloid Interface Sci..

[3] Thiruvengadam M., Chi H.Y., Kim S.-H. (2024). Impact of nanopollution on plant growth, photosynthesis, toxicity, and metabolism in the agricultural sector: An updated review. Plant Physiol. Biochem..

[4] Joyce P., Allen C.J., Alonso M.J., Ashford M., Bradbury M.S., Germain M., Kavallaris M., Langer R., Lammers T., Peracchia M.T. (2024). A translational framework to DELIVER nanomedicines to the clinic. Nat. Nanotechnol..

[5] Paramasivam G., Sanmugam A., Palem V.V., Sevanan M., Sairam A.B., Nachiappan N., Youn B., Lee J.S., Nallal M., Park K.H. (2024). Nanomaterials for detection of biomolecules and delivering therapeutic agents in theragnosis: A review. Int. J. Biol. Macromol..

[6] Dupuis V., Cerbu C., Witkowski L., Potarniche A.-V., Timar M.C., Żychska M., Sabliov C.M. (2022). Nanodelivery of essential oils as efficient tools against antimicrobial resistance: A review of the type and physical-chemical properties of the delivery systems and applications. Drug Deliv..

[7] Guidotti-Takeuchi M., de Morais Ribeiro L.N., dos Santos F.A.L., Rossi D.A., Lucia F.D., de Melo R.T. (2022). Essential Oil-Based Nanoparticles as Antimicrobial Agents in the Food Industry. Microorganisms.

[8] Jampilek J., Kralova K. (2022). Anticancer Applications of Essential Oils Formulated into Lipid-Based Delivery Nanosystems. Pharmaceutics.

[9] Radwan I.T., Baz M.M., Khater H., Selim A.M. (2022). Nanostructured Lipid Carriers (NLC) for Biologically Active Green Tea and Fennel Natural Oils Delivery: Larvicidal and Adulticidal Activities against *Culex pipiens*. Molecules.

[10] Gugleva V., Ivanova N., Sotirova Y., Andonova V. (2021). Dermal drug delivery of phytochemicals with phenolic structure via lipid-based nanotechnologies. Pharmaceuticals.

[11] de Morais Ribeiro L.N., de Paula E., Rossi D.A., Monteiro G.P., Júnior E.C.V., Silva R.R., Franco R.R., Espíndola F.S., Goulart L.R., Fonseca B.B. (2020). Hybrid pectin-liposome formulation against multi-resistant bacterial strains. Pharmaceutics.

[12] García-Pinel B., Porras-Alcalá C., Ortega-Rodríguez A., Sarabia F., Prados J., Melguizo C., López-Romero J.M. (2019). Lipid-based nanoparticles: Application and recent advances in cancer treatment. Nanomaterials.

[13] Delshadi R., Bahrami A., Tafti A.G., Barba F.J., Williams L.L. (2020). Micro and nano-encapsulation of vegetable and essential oils to develop functional food products with improved nutritional profiles. Trends Food Sci. Technol..

[14] de Araújo D.R., de Morais Ribeiro L.N., de Paula E. (2019). Lipid-based carriers for the delivery of local anesthetics. Expert Opin. Drug Deliv..

[15] Moammeri A., Chegeni M.M., Sahrayi H., Ghafelehbashi R., Memarzadeh F., Mansouri A., Akbarzadeh I., Abtahi M.S., Hejabi F., Ren Q. (2023). Current advances in niosomes applications for drug delivery and cancer treatment. Mater. Today Bio.

[16] Yasamineh S., Yasamineh P., Ghafouri Kalajahi H., Gholizadeh O., Yekanipour Z., Afkhami H., Eslami M., Kheirkhah A.H., Taghizadeh M., Yazdani Y. (2022). A state-of-the-art review on the recent advances of niosomes as a targeted drug delivery system. Int. J. Pharm..

[17] Bhardwaj P., Tripathi P., Gupta R., Pandey S. (2020). Niosomes: A review on niosomal research in the last decade. J. Drug Deliv. Sci. Technol..

[18] Ribeiro L.N.M., da Silva G.H.R., Couto V.M., Castro S.R., Breitkreitz M.C., Martinez C.S., Igartúa D.E., Prieto M.J., de Paula E. (2020). Functional Hybrid Nanoemulsions for Sumatriptan Intranasal Delivery. Front. Chem..

[19] Pavoni L., Perinelli D.R., Bonacucina G., Cespi M., Palmieri G.F. (2020). An overview of micro-and nanoemulsions as vehicles for essential oils: Formulation, preparation and stability. Nanomaterials.

[20] Pires H.M., Bastos L.M., da Silva E.F., Fonseca B.B., Sommerfeld S., de Oliveira Junior R.J. (2024). Antimicrobial Activity of Essential-Oil-Based Nanostructured Lipid Carriers against *Campylobacter* Spp. Isolated from Chicken Carcasses. Pharmaceutics.

[21] Najjari N., Sari S., Saffari M., Kelidari H., Nokhodchi A. (2022). Formulation optimization and characterization of *Pistacia atlantica* Desf. essential oil-loaded nanostructured lipid carriers on the proliferation of human breast cancer cell line SKBR3 (in vitro studies). J. Herb. Med..

[22] Lammari N., Louaer O., Meniai A.H., Fessi H., Elaissari A. (2021). Plant oils: From chemical composition to encapsulated form use. Int. J. Pharm..

[23] de Morais Ribeiro L.N., de Paula E., Rossi D.A., Martins F.A., de Melo R.T., Monteiro G.P., Breitkreitz M.C., Goulart L.R., Fonseca B.B. (2021). Nanocarriers From Natural Lipids With In Vitro Activity Against *Campylobacter jejuni*. Front. Cell. Infect. Microbiol..

[24] Viegas C., Patrício A.B., Prata J.M., Nadhman A., Chintamaneni P.K., Fonte P. (2023). Solid Lipid Nanoparticles vs. Nanostructured Lipid Carriers: A Comparative Review. Pharmaceutics.

[25] Stefanov S.R., Andonova V.Y. (2021). Lipid Nanoparticulate Drug Delivery Systems: Recent Advances in the Treatment of Skin Disorders. Pharmaceuticals.

[26] Namiot E.D., Sokolov A.V., Chubarev V.N., Tarasov V.V., Schiöth H.B. (2023). Nanoparticles in Clinical Trials: Analysis of Clinical Trials, FDA Approvals and Use for COVID-19 Vaccines. Int. J. Mol. Sci..

[27] Thi T.T.H., Suys E.J.A., Lee J.S., Nguyen D.H., Park K.D., Truong N.P. (2021). Lipid-based nanoparticles in the clinic and clinical trials: From cancer nanomedicine to COVID-19 vaccines. Vaccines.

[28] Chatzikleanthous D., O’Hagan D.T., Adamo R. (2021). Lipid-Based Nanoparticles for Delivery of Vaccine Adjuvants and Antigens: Toward Multicomponent Vaccines. Mol. Pharm..

[29] Pimentel L.S., Bastos L.M., Goulart L.R., de Morais Ribeiro L.N. (2024). Therapeutic Effects of Essential Oils and Their Bioactive Compounds on Prostate Cancer Treatment. Pharmaceutics.

[30] Sattayakhom A., Wichit S., Koomhin P. (2023). The Effects of Essential Oils on the Nervous System: A Scoping Review. Molecules.

[31] Dongre P., Doifode C., Choudhary S., Sharma N. (2023). Botanical description, chemical composition, traditional uses and pharmacology of *Citrus sinensis*: An updated review. Pharmacol. Res. Mod. Chin. Med..

[32] Haro-González J.N., Castillo-Herrera G.A., Martínez-Velázquez M., Espinosa-Andrews H. (2021). Clove essential oil (*Syzygium aromaticum* L. myrtaceae): Extraction, chemical composition, food applications, and essential bioactivity for human health. Molecules.

[33] Pimentel L.S., Sommerfeld S., de Sousa Braga P.F., Coleto A.F., Fonseca B.B., Bastos L.M., Goulart L.R., de Morais Ribeiro L.N. (2024). Antitumor activity of essential oils-based nanostructured lipid carriers on prostate cancer cells. Int. J. Pharm..

[34] Sawatphakdee G., Yostawonkul J., Oontawee S., Rodprasert W., Sawangmake C., Kornsuthisopon C., Yata T., Tabtieang S.P., Nowwarote N., Pirarat N. (2024). Feasibility of Nanostructured Lipid Carrier Loaded with Alpha-Mangostin and Clove Oil for Canine Periodontal Therapy. Animals.

[35] Múnera-Echeverri A., Múnera-Echeverri J.L., Segura-Sánchez F. (2024). Bio-Pesticidal Potential of Nanostructured Lipid Carriers Loaded with Thyme and Rosemary Essential Oils against Common Ornamental Flower Pests. Colloids Interfaces.

[36] Geronimo G., da Silva G.H.R., de Moura L.D., Ribeiro L.N.M., A Guilherme V., Mendonça T.C., Castro S.R., Breitkreitz M.C., de Paula E. (2021). Development of S75:R25 bupivacaine-loaded lipid nanoparticles functionalized with essential oils for treating melanoma. J. Chem. Technol. Biotechnol..

[37] Shah S., Dhawan V., Holm R., Nagarsenker M.S., Perrie Y. (2020). Liposomes: Advancements and innovation in the manufacturing process. Adv. Drug Deliv. Rev..

[38] Bedoya-Agudelo J.P., López-Carvajal J.E., Quiguanás-Guarín E.S., Cardona N., Padilla-Sanabria L., Castaño-Osorio J.C. (2024). Assessment of Antimicrobial and Cytotoxic Activities of Liposomes Loaded with Curcumin and *Lippia origanoides* Essential Oil. Biomolecules.

[39] Ben-Khalifa R., Gaspar F.B., Pereira C., Chekir-Ghedira L., Rodríguez-Rojo S. (2021). Essential oil and hydrophilic antibiotic co-encapsulation in multiple lipid nanoparticles: Proof of concept and in vitro activity against *Pseudomonas aeruginosa*. Antibiotics.

[40] Fahimnia F., Nemattalab M., Hesari Z. (2024). Development and characterization of a topical gel, containing lavender *(Lavandula angustifolia*) oil loaded solid lipid nanoparticles. BMC Complement. Med. Ther..

[41] Moghadam R.N., Majdizadeh M., Golbashy M., Haghiralsadat F., Hemati M. (2024). Laboratory study: Synthesis and optimization of nano nisomes containing *Bunium persicum* essential oil and investigating its toxicity on *Trichomonas vaginalis* parasite and HFF cell line. Heliyon.

[42] Zhang Z., Fu X., Wang Y., Wang J., Feng S., Zhao Z., Zheng L., Zhang J., Zhang X., Peng Y. (2023). In vivo anti-hepatitis B activity of *Artemisia argyi* essential oil-loaded nanostructured lipid carriers. Study of its mechanism of action by network pharmacology and molecular docking. Phytomedicine.

[43] da Silva E.F., Bastos L.M., Fonseca B.B., Ribas R.M., Sommerfeld S., Pires H.M., dos Santos F.A.L., de Morais Ribeiro L.N. (2024). Lipid nanoparticles based on natural matrices with activity against multidrug resistant bacterial species. Front. Cell. Infect. Microbiol..

[44] Cecchini M., Paoloni C., Campra N., Picco N., Grosso M., Perez M.S., Alustiza F., Cariddi N., Bellingeri R. (2021). Nanoemulsion of *Minthostachys verticillata* essential oil. In-vitro evaluation of its antibacterial activity. Heliyon.

[45] de Barros D.P.C., Reed P., Alves M., Santos R., Oliva A. (2021). Biocompatibility and antimicrobial activity of nanostructured lipid carriers for topical applications are affected by type of oils used in their composition. Pharmaceutics.

[46] Rodenak-Kladniew B., Castro M.A., Gambaro R.C., Girotti J., Cisneros J.S., Viña S., Padula G., Crespo R., Castro G.R., Gehring S. (2023). Cytotoxic Screening and Enhanced Anticancer Activity of *Lippia alba* and *Clinopodium nepeta* Essential Oils-Loaded Biocompatible Lipid Nanoparticles against Lung and Colon Cancer Cells. Pharmaceutics.

[47] Abedinpour N., Ghanbariasad A., Taghinezhad A., Osanloo M. (2021). Preparation of Nanoemulsions of *Mentha piperita* Essential Oil and Investigation of Their Cytotoxic Effect on Human Breast Cancer Lines. Bionanoscience.

[48] Rad E.Y., Tabrizi M.H., Ardalan P., Seyedi S.M.R., Yadamani S., Zamani-Esmati P., Sereshkeh N.H. (2020). *Citrus lemon* essential oil nanoemulsion (CLEO-NE), a safe cell-depended apoptosis inducer in human A549 lung cancer cells with anti-angiogenic activity. J. Microencapsul..

[49] Zhang X., Guo X., Sun J., Chen Y., Zhang M., Tang X., Wang W., Simal-Gandara J., Xu H., Li N. (2024). Evaluating the hypolipidemic effect of garlic essential oil encapsulated in a novel double-layer delivery system. Colloids Surf. B Biointerfaces.

[50] Souto E.B., Severino P., Marques C., Andrade L.N., Durazzo A., Lucarini M., Atanasov A.G., El Maimouni S., Novellino E., Santini A. (2020). *Croton argyrophyllus* Kunth essential oil-loaded solid lipid nanoparticles: Evaluation of release profile, antioxidant activity and cytotoxicity in a neuroblastoma cell line. Sustainability.

[51] Sriramavaratharajan V., M-Thirusenthilarasan I., Nirupama R., Vadivel V., Pragadheesh V.S., Sundaresan V., Murugan R. (2024). Volatile profiling of *Cinnamomum heyneanum* and *Cinnamomum palghatensis* and in vitro and in silico antidiabetic activity of essential oil nanoemulsions. Pharmacol. Res. Nat. Prod..

[52] Kreutz T., Lucca L.G., Carneiro S.B., Limberger R.P., Veiga-Junior V.F., de Araújo B.V., Teixeira H.F., Koester L.S. (2023). Hydrogel-thickened nanoemulsion containing amazonian *Aniba canelilla* (Kunth) Mez essential oil: Skin permeation and in vivo anti-inflammatory efficacy. J. Drug Deliv. Sci. Technol..

[53] Scuteri D., Cassano R., Trombino S., Russo R., Mizoguchi H., Watanabe C., Hamamura K., Katsuyama S., Komatsu T., Morrone L.A. (2021). Development and translation of nanobeo, a nanotechnology-based delivery system of bergamot essential oil deprived of furocumarins, in the control of agitation in severe dementia. Pharmaceutics.

[54] Santos J.S., Galvão J.G., Mendonça M.R., Costa A.M., Silva A.R., Oliveira D.S., Santos A.d.J., Lira A.A.M., Scher R., Júnior P.A.S. (2024). Encapsulation of *Citrus sinensis* essential oil and R-limonene in lipid nanocarriers: A potential strategy for the treatment of leishmaniasis. Int. J. Pharm..

[55] Ghanbariasad A., Valizadeh A., Ghadimi S.N., Fereidouni Z., Osanloo M. (2021). Nanoformulating *Cinnamomum zeylanicum* essential oil with an extreme effect on *Leishmania tropica* and *Leishmania major*. J. Drug Deliv. Sci. Technol..

[56] Siyadatpanah A., Norouzi R., Mirzaei F., Haghirosadat B.F., Nissapatorn V., Mitsuwan W., Nawaz M., Pereira M.L., Hosseini S.A., Montazeri M. (2023). Green synthesis of nano-liposomes containing *Bunium persicum* and *Trachyspermum ammi* essential oils against *Trichomonas vaginalis*. J. Microbiol. Immunol. Infect..

[57] Wen M.M., Abdelwahab I.A., Aly R.G., El-Zahaby S.A. (2021). Nanophyto-gel against multi-drug resistant *Pseudomonas aeruginosa* burn wound infection. Drug Deliv..

[58] Palmas L., Aroffu M., Petretto G.L., Escribano-Ferrer E., Díez-Sales O., Usach I., Peris J.-E., Marongiu F., Ghavam M., Fais S. (2020). Entrapment of *citrus limon* var. *Pompia* essential oil or pure citral in liposomes tailored as mouthwash for the treatment of oral cavity diseases. Pharmaceuticals.

[59] Maocha I., Rosado B., Lopes-Nunes J., Lopes M., Rolo J., Pires B., Gallardo E., Palmeira-De-Oliveira A., Martinez-De-Oliveira J., de Oliveira R.P. (2024). Imiquimod-Loaded Nanosystem for Treatment Human Papillomavirus-Induced Lesions. Pharmaceutics.

[60] Ribeiro R., Fernandes L., Costa R., Cavaleiro C., Salgueiro L., Henriques M., Rodrigues M.E. (2022). Comparing the effect of *Thymus* spp. essential oils on *Candida auris*. Ind. Crops Prod..

[61] Kowalczyk A. (2024). Essential Oils against *Candida auris*—A Promising Approach for Antifungal Activity. Antibiotics.

[62] Salam A.M.A.E., Tahoun A., Hanafy N.A. (2023). Evaluation of liposomal hydrocolloidal NPs loaded by tea tree oil as antifungal agent in vitro and in vivo investigations: Preclinical studies. Food Hydrocoll. Health.

[63] de Alteriis E., Maione A., Falanga A., Bellavita R., Galdiero S., Albarano L., Salvatore M.M., Galdiero E., Guida M. (2022). Activity of Free and Liposome-Encapsulated Essential Oil from *Lavandula angustifolia* against Persister-Derived Biofilm of *Candida auris*. Antibiotics.

[64] Baldim I., Paziani M.H., Barião P.H.G., von Zeska Kress M.R., Oliveira W.P. (2022). Nanostructured Lipid Carriers Loaded with *Lippia sidoides* Essential Oil as a Strategy to Combat the Multidrug-Resistant *Candida auris*. Pharmaceutics.

[65] Hussein A., Abdel-Mottaleb M.M., El-Assal M., Sammour O. (2020). Novel biocompatible essential oil-based lipid nanocapsules with antifungal properties. J. Drug Deliv. Sci. Technol..

[66] Gross I.P., Lima A.L., Sousa E.C., Souza M.S., Cunha-Filho M., da Silva I.C.R., Orsi D.C., Sá-Barreto L.L. (2024). Antimicrobial and acaricide sanitizer tablets produced by wet granulation of spray-dried soap and clove oil-loaded microemulsion. PLoS ONE.

[67] Drais H.K. (2023). Development and Evaluation Essential Oils Nanoemulgel as Human Skin Sanitizer Using Novel Method. Turk. J. Pharm. Sci..

[68] Mechmechani S., Khelissa S., Gharsallaoui A., Omari K.E., Hamze M., Chihib N.-E. (2022). Hurdle technology using encapsulated enzymes and essential oils to fight bacterial biofilms. Appl. Microbiol. Biotechnol..

[69] Unnisa A., Chettupalli A.K., Alazragi R.S., Alelwani W., Bannunah A.M., Barnawi J., Amarachinta P.R., Jandrajupalli S.B., Elamine B.A., Mohamed O.A. (2023). Nanostructured Lipid Carriers to Enhance the Bioavailability and Solubility of Ranolazine: Statistical Optimization and Pharmacological Evaluations. Pharmaceuticals.

[70] Dumlu B. (2024). Importance of Nano-Sized Feed Additives in Animal Nutrition. J. Agric. Prod..

[71] Gelaye Y. (2024). Application of nanotechnology in animal nutrition: Bibliographic review. Cogent Food Agric..

[72] Meligy A.M., El-Hamid M.I.A., Yonis A.E., Elhaddad G.Y., Abdel-Raheem S.M., El-Ghareeb W.R., Mohamed M.H., Ismail H., Ibrahim D. (2023). Liposomal encapsulated oregano, cinnamon, and clove oils enhanced the performance, bacterial metabolites antioxidant potential, and intestinal microbiota of broiler chickens. Poult. Sci..

[73] de Souza Silveira Valente J., de Oliveira da Silva Fonseca A., Brasil C.L., Sagave L., Flores F.C., de Bona da Silva C., Sangioni L.A., Pötter L., Santurio J.M., de Avila Botton S. (2016). In Vitro Activity of *Melaleuca alternifolia* (Tea Tree) in Its Free Oil and Nanoemulsion Formulations Against *Pythium insidiosum*. Mycopathologia.

[74] Qadeer A., Khan A., Khan N.M., Wajid A., Ullah K., Skalickova S., Chilala P., Slama P., Horky P., Alqahtani M.S. (2024). Use of nanotechnology-based nanomaterial as a substitute for antibiotics in monogastric animals. Heliyon.

[75] Choudhary P., Bhanjana G., Kumar S., Dilbaghi N. (2024). Development and evaluation of eco-friendly carvacrol nanoemulsion as a sustainable biopesticide against bacterial leaf blight of cluster bean. Pest. Manag. Sci..

[76] Kaur R., Choudhary D., Bali S., Bandral S.S., Singh V., Ahmad A., Rani N., Singh T.G., Chandrasekaran B. (2024). Pesticides: An alarming detrimental to health and environment. Sci. Total Environ..

[77] Luker H.A., Salas K.R., Esmaeili D., Holguin F.O., Bendzus-Mendoza H., Hansen I.A. (2023). Repellent efficacy of 20 essential oils on *Aedes aegypti* mosquitoes and Ixodes scapularis ticks in contact-repellency assays. Sci. Rep..

[78] Pereira P.C., Parente C.E., Carvalho G.O., Torres J.P., Meire R.O., Dorneles P.R., Malm O. (2021). A review on pesticides in flower production: A push to reduce human exposure and environmental contamination. Environ. Pollut..

[79] Pathak A., Mandal N., Upadhyaya D.C., Joshi N., Upadhyaya C.P. (2024). Lipid nanoparticles: A sustainable solution for crop disease management. Adv. Nat. Sci. Nanosci. Nanotechnol..

[80] Hikal W.M., Baz M.M., Alshehri M.A., Bahattab O., Baeshen R.S., Selim A.M., Alhwity L., Bousbih R., Alshourbaji M.S., Ahl H.A.H.S.-A. (2023). Sustainable Pest Management Using Novel Nanoemulsions of Honeysuckle and Patchouli Essential Oils against the West Nile Virus Vector, *Culex pipiens*, under Laboratory and Field Conditions. Plants.

[81] Rodrigues A.B.L., Martins R.L., de Menezes Rabelo É., Tomazi R., Santos L.L., Brandão L.B., Faustino C.G., Farias A.L.F., dos Santos C.B.R., de Castro Cantuária P. (2021). Development of nano-emulsions based on *Ayapana triplinervis* essential oil for the control of *Aedes aegypti* larvae. PLoS ONE.

[82] Abdel-Ghany H.S.M., Abdel-Shafy S., Abuowarda M., El-Khateeb R.M., Hoballah E.M., Fahmy M.M. (2021). Acaricidal activity of *Artemisia herba-alba* and *Melia azedarach* oil nanoemulsion against *Hyalomma dromedarii* and their toxicity on Swiss albino mice. Exp. Appl. Acarol..

[83] Palermo D., Giunti G., Laudani F., Palmeri V., Campolo O. (2021). Essential oil-based nano-biopesticides: Formulation and bioactivity against the confused flour beetle *tribolium confusum*. Sustainability.

[84] Dunan L., Malanga T., Benhamou S., Papaiconomou N., Desneux N., Lavoir A.-V., Michel T. (2023). Effects of essential oil-based formulation on biopesticide activity. Ind. Crops Prod..

[85] Manjesh K., Kundu A., Dutta A., Saha S., Neelakanthaiah B.S. (2022). Bio-Insecticidal Nanoemulsions of Essential Oil and Lipid-Soluble Fractions of *Pogostemon cablin*. Front. Plant Sci..

[86] Teng Q., Li Y., Cai Y., Guo J., Zou M., Xue Q., Tang X., Li X., Zhao J. (2024). A potential acaricide of *Moutan cortex* essential oil encapsulated in nanoemulsion and mesoporous silica nanoparticles against the house dust mite *Dermatophagoides farinae*. J. Pest Sci..

[87] Barbhuiya R.I., Wroblewski C., Elsayed A., Subramanian J., Kaur G., Routray W., Singh A. (2024). Development and physicochemical characterization of *Azadirachta indica* seed oil loaded niosomes nanoparticles: A potential natural pesticide. Chem. Eng. Res. Des..

[88] Adamu A., Ahmad K., Siddiqui Y., Ismail I.S., Asib N., Bashir Kutawa A., Adzmi F., Ismail M.R., Berahim Z. (2021). Ginger essential oils-loaded nanoemulsions: Potential strategy to manage bacterial leaf blight disease and enhanced rice yield. Molecules.

[89] Tortorici S., Cimino C., Ricupero M., Musumeci T., Biondi A., Siscaro G., Carbone C., Zappalà L. (2022). Nanostructured lipid carriers of essential oils as potential tools for the sustainable control of insect pests. Ind. Crops Prod..

[90] Almeida L.F., Gil G.A., Moraes L.N., Furtado F.B., Kakuda L., Grotto R.M.T., Oliveira W.P. (2024). Nanostructured lipid carriers loaded with essential oils: A strategy against SARS-CoV-2. J. Microencapsul..

[91] Shehabeldine A.M., Doghish A.S., El-Dakroury W.A., Hassanin M.M.H., Al-Askar A.A., AbdElgawad H., Hashem A.H. (2023). Antimicrobial, Antibiofilm, and Anticancer Activities of *Syzygium aromaticum* Essential Oil Nanoemulsion. Molecules.

[92] Ekrami A., Ghadermazi M., Ekrami M., Hosseini M.A., Emam-Djomeh Z., Hamidi-Moghadam R. (2022). Development and evaluation of *Zhumeria majdae* essential oil-loaded nanoliposome against multidrug-resistant clinical pathogens causing nosocomial infection. J. Drug Deliv. Sci. Technol..

[93] Tazehjani D.A.J., Farahpour M.R., Hamishehkar H. (2021). Effectiveness of topical caraway essential oil loaded into nanostructured lipid carrier as a promising platform for the treatment of infected wounds. Colloids Surf. A Physicochem. Eng. Asp..

[94] Aguilar-Pérez K.M., Medina D.I., Narayanan J., Parra-Saldívar R., Iqbal H.M.N. (2021). Synthesis and nano-sized characterization of bioactive oregano essential oil molecule-loaded small unilamellar nanoliposomes with antifungal potentialities. Molecules.

[95] Usach I., Margarucci E., Manca M.L., Caddeo C., Aroffu M., Petretto G.L., Manconi M., Peris J.-E. (2020). Comparison between citral and pompia essential oil loaded in phospholipid vesicles for the treatment of skin and mucosal infections. Nanomaterials.

[96] Khezri K., Farahpour M.R., Mounesi Rad S. (2020). Efficacy of *Mentha pulegium* essential oil encapsulated into nanostructured lipid carriers as an in vitro antibacterial and infected wound healing agent. Colloids Surf. A Physicochem. Eng. Asp..

[97] Hamedi H., Moradi S., Tonelli A.E., Hudson S.M. (2019). Preparation and characterization of chitosan–Alginate polyelectrolyte complexes loaded with antibacterial thyme oil nanoemulsions. Appl. Sci..

[98] Ghodrati M., Farahpour M.R., Hamishehkar H. (2019). Encapsulation of Peppermint essential oil in nanostructured lipid carriers: In-vitro antibacterial activity and accelerative effect on infected wound healing. Colloids Surf. A Physicochem. Eng. Asp..

[99] Carbone C., Teixeira M.D.C., Sousa M.D.C., Martins-Gomes C., Silva A.M., Souto E.M.B., Musumeci T. (2019). Clotrimazole-loaded mediterranean essential oils NLC: A synergic treatment of *Candida* skin infections. Pharmaceutics.

[100] Meghani N., Mansuri A., Chaudhari R., Kumar A. (2024). Fabrication, assessment, and potential anti-bacterial activity of sandalwood oil nanoemulsion and its hand rub sanitizer. Environ. Sci. Pollut. Res..

[101] Mehta M., Bui T.A., Yang X., Aksoy Y., Goldys E.M., Deng W. (2023). Lipid-Based Nanoparticles for Drug/Gene Delivery: An Overview of the Production Techniques and Difficulties Encountered in Their Industrial Development. ACS Mater. Au.

[102] Biscaia-Caleiras M., Fonseca N.A., Lourenço A.S., Moreira J.N., Simões S. (2024). Rational formulation and industrial manufacturing of lipid-based complex injectables: Landmarks and trends. J. Control. Release.

[103] Eugster R., Orsi M., Buttitta G., Serafini N., Tiboni M., Casettari L., Reymond J.-L., Aleandri S., Luciani P. (2024). Leveraging machine learning to streamline the development of liposomal drug delivery systems. J. Control. Release.

[104] Bezelya A., Küçüktürkmen B., Bozkır A. (2023). Microfluidic Devices for Precision Nanoparticle Production. Micro.

[105] Javed S., Mangla B., Salawi A., Sultan M.H., Almoshari Y., Ahsan W. (2024). Essential Oils as Dermocosmetic Agents, Their Mechanism of Action and Nanolipidic Formulations for Maximized Skincare. Cosmetics.

[106] Chemat F., Abert-Vian M., Fabiano-Tixier A.S., Strube J., Uhlenbrock L., Gunjevic V., Cravotto G. (2019). Green extraction of natural products. Origins, current status, and future challenges. TrAC Trends Anal. Chem..

